# Overview on *Cryptosporidium bovis* and Its Effect on Calves in Some Governorates in Egypt

**DOI:** 10.1155/2022/4271063

**Published:** 2022-05-31

**Authors:** Amer R. Abdelaziz, Amin Tahoun, Hanem El-Sharkawy, Moustafa M. Abd El-Salam, Mohammed Alorabi, Ahmed M. El-Shehawi, Rasha A. El Meghanawy, Essam E Toukhy, Ahmed M. Abd El-Salam, Shimaa S. G. Sorour

**Affiliations:** ^1^Department of Parasitology, Faculty of Veterinary Medicine, Sohag University, Sohag, Egypt; ^2^Department of Animal Medicine, Faculty of Veterinary Medicine, Kafrelsheikh University, Kafrelsheikh 33511, Egypt; ^3^Department of Poultry and Rabbit Diseases, Faculty of Veterinary Medicine, Kafrelsheikh University, Kafrelsheikh 33511, Egypt; ^4^Department of Animal Production, Faculty Agriculture, Tanta University, Tanta, Egypt; ^5^Department of Biotechnology, Faculty of Science, Taif University, P.O. Box 11099, Taif 21944, Saudi Arabia; ^6^Department of Biotechnology, Animal Health Research Institute -ARC, Giza, Egypt; ^7^Department of Parasitology, Shebin El Koom Lab., Animal Health Research Institute -ARC, Giza, Egypt; ^8^Department of Fish Processing and Biotechnology, Faculty of Aquatic and Fisheries Science, Kafrelsheikh University, Kafrelsheikh 33511, Egypt; ^9^Department of Parasitology, Faculty of Veterinary Medicine, Kafrelsheikh University, Kafr el-Sheikh, Egypt

## Abstract

The present study was conducted to elucidate the prevalence of *Cryptosporidium bovis* in suckling and weaned cattle calves (*Bubalus bubalis*) from different governorates in northern, middle, and southern Egypt, such as Behera, Menofia, Qaliubiya, Assiut, and Sohag; result revealed that from the overall examined fecal samples (*n* = 825), the overall prevalence was 7.27%, the highest significant infection rate was in young suckling calves less than one month (8.2%), and seasonally, winter season has the highest significant level (11.24%), but sex and locality were of no significant effect on the prevalence of infection in this study. Gene sequencing and phylogenetic analysis of the 18SSU-rRNA gene of the local bovine isolate were performed, and it was found that *C. bovis* genotype was highly similar to human isolate, which provoke the zoonotic transmission of bovine isolate to humans and identified as a potential source for human cryptosporidiosis infection in Egypt.

## 1. Introduction

*Cryptosporidium* spp., is an obligatory intracellular protozoan of intestinal, respiratory, and gall bladder epithelia that infect several domestic and wild animals species and humans [1]. It is considered a significant public health concern due to its potential transmission through human food and drinks causing outbreak in newborns of humans and animals [2, 3]. The main most frequent *Cryptosporidium* spp. encountered in cattle are *C. ryanae*, *C. andersoni, C. parvum*, and *C. bovis* [4–6]. At the time of adaptation from umbilical to oral nutrition, calves are under intense pressure that give opportunity for serious pathogens such as *Cryptosporidium* species to colonize the intestine with clinical manifestation related only to *C. parvum* [7]. In many countries, this group of parasites was reported to be the main cause of enteritis in calves [5, 8, 9]. Of these parasites, *Cryptosporidium parvum* has been shown to have a potential high zoonotic risk and has been reported to be the most prevalent protozoan parasite distributed all over the world [8, 10–12]. It was reported to cause a high infection rate in calves with the manifestation of watery diarrhea, dehydration, slow growth rate, and low weight gain with a high mortality rate of nontreated calves, leading to a huge economic loss in the newborn' first days of the newborn [13]. Moreover, these infected new born disseminate the parasite and infect human causing outbreaks of cryptosporidiosis [14], especially the immune-compromised individuals as babies, senile, and those having AIDS [15]. Indeed, clinical cryptosporidiosis is considered a challenge to control due to the ability of a small number of *C. parvum* oocysts to experimentally colonize and multiply in the gut and cause infection and manifest its clinical disease [13, 16, 17]. The possibility of young calves being infected with *C. parvum* is high as its prevalence rate in preweaned calves was reported to be 28.0–80.0% in the UK [8, 11, 12] and 96.6% in the USA [18].

Most of the screening studies have focused on *C. parvum* for its potential zoonotic importance; meanwhile, *C. bovis* is nearly neglected in spite of its clinical and zoonotic importance. In Austria, *C. bovis* was reported to be isolated with *C. ryanae* from feces of postweaned calves [3]. These two parasites were also detected in fecal samples of healthy and diarrheic calves in Sudan, China, and Sweden [19–21]. Using the molecular method for detection, *C. bovis* was recently isolated from 7 out of 177 preweaned calves in Austria that was described to be more efficient and higher in detection than the previous method for detection [22]; for the best of the authors' knowledge, there is no exact information about the *C. bovis* prevalence in Egypt. Therefore, this study was designed with the aim of detecting the prevalence of *C. bovis* in different localities in Egypt.

## 2. Materials and Methods

### 2.1. Ethical Considerations

The study design and procedures were carefully reviewed and approved by the local guidelines on Research, Publication, and Ethics of the Faculty of Veterinary Medicine of Sohag University, Egypt, which complies with all relevant Egyptian legislation.

### 2.2. Sample Collection and Processing

**Figure 1:**
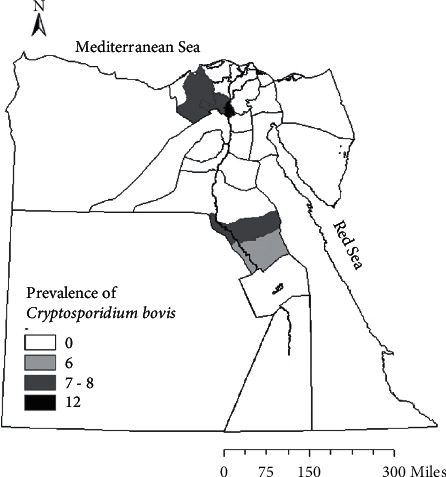
*Cryptosporidium bovis* among different governorates in Egypt.

The study area includes the Behera governorate that represents northern Egypt, Menofia and Qaliubiya that represent middle Egypt, and the southern Egypt that includes Sohag and Assiut governorates which lie on the sides of River Nile (Figure 1).

Fecal samples from natural infected young calves were collected in sterile plastic cups and labelled (date, age, sex, locality, from July 2019 till June 2020). Samples were centrifuged and preserved in 10% formalin and then sent to parasitology lab of Sohag university.

Fecal samples were stirred well and filled with saturated salt solution. The solution was centrifuged at 500x for 5 minutes; then, supernatant was removed. A drop of remaining was smeared on the microscopic glass slide.

Microscopic examination was performed using modified Ziehl–Neelsen stain (MZN) briefly; the smeared slide was transferred to a slide warmer at 60°C until dried, fixed with 96% methanol for 30 seconds, and fixed in flame for a few seconds. The slide then stained with Kinyoun's carbol fuchsin for 20–25-minutes, rinsed with distilled water, drained, and destained with acid alcohol for 2 minutes, rinsed with distilled water, drained, and stained with malachite green for 10–15 minutes, and then rinsed with distilled water, drained, and dried on a slide warmer at 60°C for about 5 minutes. Mounting was done with a coverslip using Canada balsam and examined using the light microscope with 100x oil immersion objective [23].

### 2.3. DNA Extraction

Positive fecal samples from each governorate were included and pooled to obtain concentrated oocytes of *Cryptosporidium* sp. by Sheather's sugar flotation (Sheather 1923). DNA was extracted using the QIAamp DNA Stool Mini Kit (Qiagen Inc., Valencia, CA) according to the detailed manufacturer's instructions. Briefly, 2 ml of fasces was centrifuged at 2500 rpm for 5 minutes at 4°C. 200 *µ*l of PBS was added to the precipitate and resuspended completely by pipetting. Then, 20 *µ*l of proteinase K and 2 *µ*l of lysis enhancer was added to the samples and mixed immediately. Finally, 200 *µ*l of buffer TB was mixed well by pulsed vortex and incubated for 10 min at 65°C [24].

### 2.4. Conventional PCR

PCR amplification of extracted oocyst DNA was done using the 18SSU-rRNA target gene of *Cryptosporidium* sp. specific primers, 1 *µ*l from each primer (forward: Crypto F.5p (5′-AACCTGGTTGATCCTGCCAGTAGTC-3′) and reverse: Crypto R.5p (5′-TGATCCTTCTGCAGGTTCACCTACG-3)) [25], and 6 *µ*l of PCR water (DNA free water) to make the final volume 50 *µ*l, and the mixture was amplified in the DNA thermal cycler (Rotor-Gene Q: QIAGEN). A total 45 cycles were performed, each consisting of 1 minute of denaturation at 94°C, 2 minutes of annealing at 50°C, and 3 minutes of extension at 72°C with a preliminary step at 95°C for 5 minutes. In a 1.5% agarose gel, PCR products were stained with ethidium bromide and evaluated under UV light using an image documentation system. The positive sample was identified by the presence of the predicted band size in the gel [26].

### 2.5. Gene Sequencing and Phylogenetic Analysis

The PCR products were purified and pooled for sequencing using a QIAquick PCR purification kit (Qiagen), DNA was concentrated by vacuum centrifuge and stored at −20°C until sequencing was performed [27], the selected purified amplicon was pooled and cycle sequenced at Molecular Biology Unit, Assiut University, Egypt, the using ABI 377-automated DNA Big Dye Terminator v3.1 Cycle Sequencing Ready Reaction Kit (Applied Biosystems Inc., Foster City, CA, USA) on the automated capillary sequencer (ABI Prism 3100, Applied Biosystems) using the same primers. The sequences were assembled using DNASTAR module SeqMan II. The Bootstrap MP tree was inferred using 1.000 replicates, and phylogenetic analysis was performed using maximum likelihood, neighbor joining, and maximum parsimony in MEGA7 [28].

### 2.6. Statistical Analysis

Fisher's exact test and odds ratio (OR) with 95% confidence intervals (95% CI) were analysed using GraphPad Prism software version 6 (GraphPad Software, Inc., La Jolla, CA, USA) in order to compute the categorical variables included in this study. A *P* value <0.05 was considered significant.

## 3. Results

**Figure 2:**
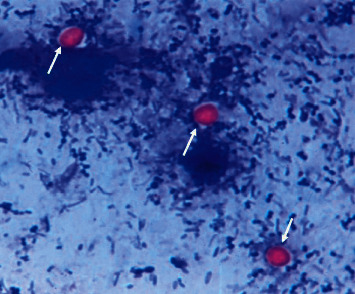
Oocyst of *Cryptosporidium* sp. (arrows) 100x stained using modified Ziehl–Neelsen stain.

The microscopic examination of stained smears confirms the presence of *Cryptosporidium* sp. which appeared as pink spherical bodies in blue background (Figure 2, 100x).

**Figure 3:**
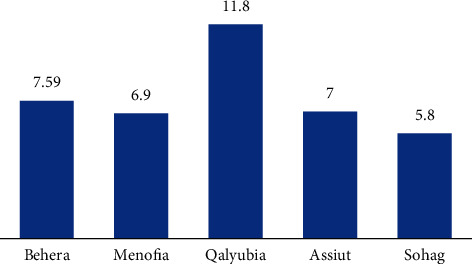
Prevalence of *Cryptosporidium bovis* infection of calves among different governorates of Egypt.

**Figure 4:**
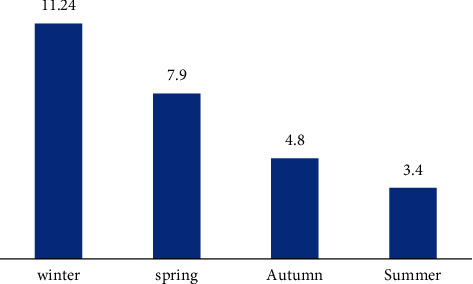
Seasonal variation of *Cryptosporidium bovis* infection in calves in Egypt.

**Figure 5:**
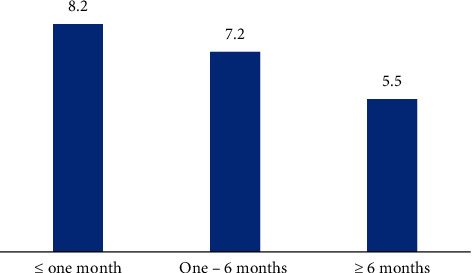
Variation in age of *Cryptosporidium bovis* infection of calves in Egypt.

The overall prevalence of infection was 7.27% as 60 calves were found to be infected with the etiological agent out of 825 calves from the five governorates in northern and middle Egypt (Behera, Menofia, and Qaliubiya) and southern Egypt (Assiut and Sohag); the highest infection rate was found in Qaliubiya (11.8%), followed by the governorate of Behera (7.59%), and the lowest percent of infection was found in Sohag (5.8%), and this variation in the infection rate in relation to localities was not significant (*P* value ≤0.05) (Table 1), as shown in Figures 1 and 3. Seasonal variation, as shown in Figure 4, in this study, had a highly significant effect on the infection rate (*P* value ≤0.05); at winter season, calves showed the highest significant infection rate (11.24%), followed by spring (7.9%), and the lowest infection rate was prevailed in the summer season (3.4%); the *P* value was (0.021), significant 95%, and the odd ratio (1.09) and confidence interval between (0.987–1.198), for the age group as shown in Figure 5; the highest significant infection rate (*P* value ≤0.05) was found in suckling calves from birth to one month old (8.2%), followed by calves aged from 1 to 6 months (7.2%). The lowest prevalence rate was found among weaned calves over 6 months (5.5%). The odds ratio was estimated at 1.02 (95% CI: 0.977–1.083), which was not significant. Also, it was found that the infection rate in female calves (7.49%) was the same as the males (7.03%) (*P* value ≤0.05).

**Figure 6:**
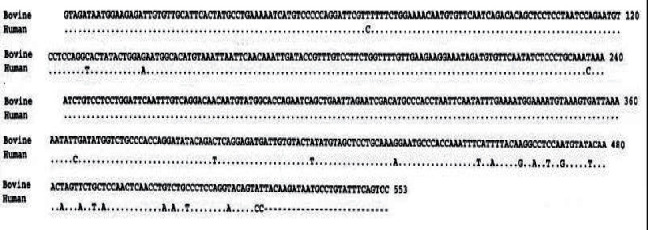
Gene sequencing of *Cryptosporidium parvum* bovine isolate using the 18SSU-rRNA target gene and it showed a high identity to human isolate 97.8%.

The results of molecular characterization, gene sequencing, and phylogenetic analysis using 18SSU-rRNA as target gene revealed bands of about 452 bp which was similar to human samples of about 456 bp. Gene sequencing showed a length of 553 nucleotides, (C + G = 233), (A + T = 320), and the frequency of the nucleotides was as follows: adenine (A): 0.276, cytosine (C): 0.250, guanine (G): 0.203, and thymine (T): 0.271. The alignments of resulted sequence with *C. parvum* human isolate were identical (97.8%) which is considered analogous to *C. parvum* IIa (Figure 6).

**Figure 7:**
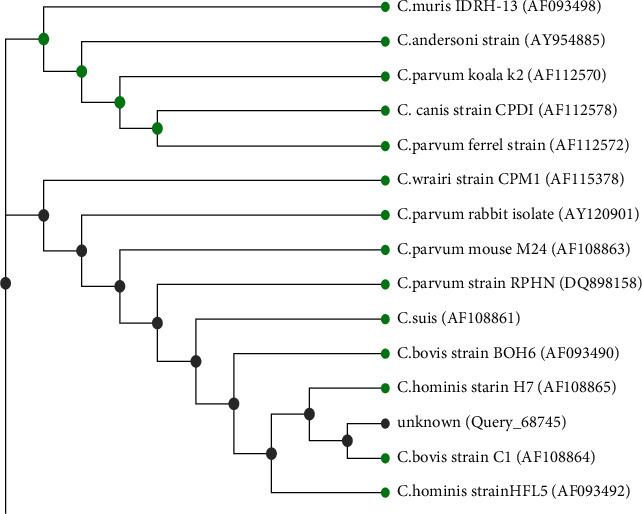
Phylogenetic maximum likelihood tree of the 18SSU-rRNA gene sequences with 1000 repeats Bootstrap. The sequence of this study (Query_68745) was aligned with 14 other sequences of human and animal isolates of *Cryptosporidium* species from GenBank.

The phylogenetic tree analysis was performed by applying Neighbor-Join analysis to a matrix of pairwise distances estimated using the maximum composite likelihood (MCL) approach. Analysis showed that there were a total of 553 positions in the final dataset, and it was found that the isolate of the present study was on the same branch and was identical (99%) in a similarity rate to *C. bovis* strain *C1* (*AF108864*) and also (97.8%) similar to human strain of *C. hominis HFL5* (*AF093492*) and 98% similar with *C. hominis* strain *H7* (*AF108865*), which proved the zoonotic impact of bovine *Cryptosporidium* spp., Egyptian strain (Figure 7). Evolutionary analyzes were conducted in MEGA7.

## 4. Discussion

The detection of *Cryptosporidium* infection in fecal samples of human and animals is accomplished by different tools and techniques [29]. The direct staining method of fecal samples by modified Ziehl–Nilsen (MZN) was conducted in this study to screen positive samples of *Cryptosporidium* oocysts; this technique is the main tool which is recommended as a screening method in epidemiological studies by [30] due to its accurate and précised results, but the main defect in this technique is the difficulty to discriminate between different species of *Cryptosporidium* depending only on morphology and morphometry [31]. In the present study, the overall prevalence was 7.27%, by microscopy using MZN stain as a screening tool, which is apparently low in comparison to other parasitic infection, and it may be due to the regular treatment program provided to young animals by farmers and veterinarians in selected localities; the incidence rate of *Cryptosporidium* all over the world ranged from 3.4 to 96.6% which has been reported in calves as previously reviewed [13]; this wide variation in infection occurrence may be due to geographical distribution (study location), changes in climates, and management practices in the farm and also may be as a relation to design of the study (samples number, season, and the diagnostic technique); this variation in occurrence is affected by these factors which affect the transmission of *Cryptosporidium* between animals [32–34].

In the current study, we reported a strong significant correlation between the season of sample collection and the occurrence, and it was found that the winter cold season of Egypt was highly suitable for the reproduction and shedding of oocyst in fecal samples; it may be due to the partial immunosuppression of neonates and preweaned calves and winter cold climate; this due to most parturition cases happened at winter in Egypt; on the other hand, partial immunosuppression is due to the fact that most cattle owners and farmers remove calves from colostrum and sell them in markets, which lead to decreased immunity, and *Cryptosporidium* develops, and so, the most affected age groups were the calves from birth till one month; it was of the highest percent of infection among age groups; this was in accordance with [35], and it was observed that with advance in age, the infection was lower; this may be due to when the intestinal epithelium is severely damaged by *Cryptosporidium* infection, parasitic reproduction is impaired, so the shedding is decreased with age and advance of infection, and this statement was in agreement with [36], who stated that four common *Cryptosporidium* species have been identified in cattle: *C. parvum*, *C. bovis*, *C. ryanae*, and *C. andersoni*, but only *C. bovis* is associated with clinical disease in neonatal calves, as older animals (>6 weeks) exhibit asymptomatic oocyst shedding. A recent study by [37] on *C. hominis* in symptomatic and asymptomatic calves in France proved the importance of identifying which *Cryptosporidium* species are shed by calves. Species-specific multiplex PCR or real-time PCR could be used to detect low-level infections as mentioned [38]. Current guidelines suggest that genetic characterization of *Cryptosporidium* isolates should be based on genetic loci conserved *18S rRNA* gene [39]; thus, in our study, the *18S rRNA* was targeted, concordant results were obtained for the majority of isolates, and sequence analysis of the *18S rRNA* gene showed that four *C. parvum* IIa subgenotype groups exist: (IIaA15G2R1), (IIaA17G3R1), (IIaA17G1R1), and (IIaA19G1R1). These results were in agreement with other studies which found that *C. parvum* IIa is the common subtype family in humans and calves [40]. Thus, this subtype family is considered to be potentially zoonotic and transmissible from livestock (especially young calves) as stated by [41]; thus, this study elucidated the zoonotic impact of calf as a reservoir of infection with cryptosporidiosis, and other future research works are mandatory to detect if this subtype provoke a high morbidity and to what extent, and similar studies must be on a larger geographic area with greater numbers and are needed to increase our understanding of cryptosporidiosis biology and life cycle dynamics in calves.

## 5. Conclusions

This study concluded that preweaned suckling calves from birth till one month are the main risk factors for infection with *Cryptosporidium bovis*, and cold winter season is the highly significant potential risk factor for infection with cryptosporidiosis in calves. The specific isolate in this study is of high zoonotic impact on humans, which is highly similar to the human strain of *Cryptosporidium* sp., and thus, young calves are considered the main zoonotic reservoir for human infection with cryptosporidiosis.

## Figures and Tables

**Table 1:** Detection of *Cryptosporidium* sp. using modified Ziehl–Neelsen staining of samples that have been collected and prevalence of *Cryptosporidium* sp., in calves among different governorates of Egypt (locality, season, age, and sex).

Locality	Exam.	Inf.	%	Odds ratio (95% CI)	*P* value (≤0.05)
Behera	237	18	7.59	0.89 (0.887–0.998)	0.0611
Menofia	115	8	6.9		
Qaliubiya	76	9	11.8		
Assiut	157	11	7		
Sohag	240	14	5.8		
Total	825	60	**7.27**

Season	Exam.	Inf.	%	Odds ratio (95% CI)	*P* value (≤0.05)

Winter	249	28	11.24	1.09 (0.987–1.198)	0.021
Spring	215	17	7.9		
Autumn	186	9	4.8		
Summer	175	6	3.4		
Total	825	60	**7.27**

Age	Exam.	Inf.	%	Odds ratio (95% CI)	*P* value (≤0.05)

≤ One month	338	28	8.2	1.02 (0.977–1.083)	0.0262
1–6 months	289	21	7.2		
≥6 months	198	11	5.5		
Total	825	60	**7.27**

Sex	Exam.	Inf.	%	Odds ratio (95% CI)	*P* value (≤0.05)

Male	398	28	7.03	0.92 (0.877–0.98)	0.0562
Female	427	32	7.49		
	
Total	825	60	**7.2**7

Bold values indicate the total percentage of infected animals.

## Data Availability

The data used to support the findings of this study are available from the corresponding author upon request.
